# A new early Eocene deperetellid tapiroid illuminates the origin of Deperetellidae and the pattern of premolar molarization in Perissodactyla

**DOI:** 10.1371/journal.pone.0225045

**Published:** 2019-11-08

**Authors:** Bin Bai, Jin Meng, Fang-Yuan Mao, Zhao-Qun Zhang, Yuan-Qing Wang

**Affiliations:** 1 Key Laboratory of Vertebrate Evolution and Human Origins of Chinese Academy of Sciences, Institute of Vertebrate Paleontology and Paleoanthropology, Chinese Academy of Sciences, Beijing, China; 2 CAS Center for Excellence in Life and Paleoenviroment, Beijing, China; 3 State Key Laboratory of Palaeobiology and Stratigraphy, Nanjing Institute of Geology and Palaeontology, Chinese Academy of Sciences, Nanjing, China; 4 Division of Paleontology, American Museum of Natural History, New York, United States of America; 5 Earth and Environmental Sciences, Graduate Center, City University of New York, New York, United States of America; 6 College of Earth and Planetary Sciences, University of Chinese Academy of Sciences, Beijing, China; Royal Belgian Institute of Natural Sciences, BELGIUM

## Abstract

Deperetellidae is a clade of peculiar, Asian endemic tapiroids from the early and middle Eocene. The previously published material mainly comprises maxillae, mandibles, and some postcranial elements. However, the absence of cranial materials and primitive representatives of the deperetellids obscures their phylogenetic relationships within Tapiroidea. Furthermore, derived deperetellids have completely molarized premolars, but the pattern of their evolution remains unclear. Here, we report a nearly complete skull and some carpals of a new basal deperetellid tapiroid, *Irenolophus qii* gen. et sp. nov., from the late early Eocene of the Erlian Basin, Inner Mongolia, China. We suggest that deperetellids (along with Tapiridae) probably also arose from some basal ‘helaletids’, based on the reduced, flat, lingually depressed metacones on the upper molars, the trend towards the bilophodonty on the lower molars, and a shallow narial notch with the premaxilla in contact with the nasal. The molarization of the premolars in Deperetellidae from *Irenolophus* through *Teleolophus* to *Deperetella* was initiated and gradually enhanced by the separation between the paraconule and the protocone. That pattern differs from the protocone-hypocone separation in helaletids, tapirids, and most rhinoceroses, and the metaconule-derived pseudohypocone in amynodontids. However, the specific relationship of deperetellids within Tapiroidea and the roles of different patterns of premolar molarization in perissodactyl evolution need further and comprehensive study.

## Introduction

Deperetellidae is a group of endemic Asian tapiroids known from Eocene deposits in China, Mongolia, Kyrgyzstan, and Myanmar [1–3]. Deperetellids are peculiar among tapiroids in possessing an inverted U-shaped ridge on the upper molar [1], cement on the cheek teeth [4], a Hunter-Schreger Bands with compound configuration [5], and postcranial adaptations for fast running [1, 6]. The known specimens of deperetellids are composed mainly of maxillae, mandibles, and a few postcranial elements [1, 6]. Recently, Bai et al. [6] described additional postcranial morphologies of *Teleolophus* ? *medius* from the Ulan Shireh Formation of the Erlian Basin, China. Although deperetellid fossils are relatively abundant and common in the Eocene Asian deposits, their phylogenetic relationships with other tapiroids have long been remained controversial.

Here, we report a nearly complete skull and some carpals of a new primitive deperetellid from the late early Eocene of the basal Arshanto Formation of the Erlian Basin, Inner Mongolia, China, which sheds light on the origin of deperetellids and their phylogenetic position among tapiroids. We also review the previously reported deperetellids from the Arshanto Formation. Furthermore, on the basis of the evolutionary lineage from the new primitive deperetellid through *Teleolophus* to *Deperetella*, we propose a special premolar molarization process through the separation of the paraconule and protocone in Deperetellidae, and discuss the distribution of this pattern in other perissodactyls. Moreover, two other patterns of premolar molarization in perissodactyls also are investigated and compared with that of deperetellids.

## Methods

### Morphological comparisons

We compared the new cranial material with that of the helaletid *Heptodon* [7], *Colodon* [8], and the lophialetid *Lophialetes* [9], because helaletids and lophialetids are considered to be closely related to deperetellids [10, 11]. We also compared the dentition and carpals with those of the more derived deperetellids *Teleolophus* and *Deperetella* [1, 6], the helaletids *Heptodon* [7] and *Colodon* [12, 13], and the lophialetid *Lophialetes* [1].

### Enamel microstructure

A partial m2 talonid of *Irenolophus* sp. (IVPP V 25832) was embedded in DPX mounting medium used for the enamel microstructure analysis. The prepared specimen was cut in cross, vertical, and tangential sections. Samples were observed using a binocular polarizing light microscope and digital images were captured using a Canon digital camera with a macro lens and a Zeiss microscope (SteREO Discovery V. 20) with a digital imaging system (AxioVision SE64 Rel. 4.9). Some sections were subsequently etched with 0.1 mol/L phosphoric acid for about 90 seconds. After rinsing and air-drying, the sample was examined with a Zeiss MAEV025 scanning electron microscope. Most SEM photographs were taken at a voltage of 3 kVwith magnifications commonly between 500 − 5000X to observe the three-dimensional enamel prism orientation, prism shape, and crystallite structures. Most optical photographs were taken with magnifications under 20X to identify the Hunter-Schreger Bands (HSB) and Schmelzmuster. The appearance of the HSB was observed in variable light following the method of Koenigswald et al. [5]. All facilities are in the Key Laboratory of Vertebrate Evolution and Human Origins, IVPP, Chinese Academy of Sciences.

### Institutional abbreviations

**AMNH FM**, American Museum of Natural History, Fossil Mammals, New York; **IVPP**, Institute of Vertebrate Paleontology and Paleoanthropology, Beijing.

### Nomenclatural act

The electronic edition of this article conforms to the requirements of the amended International Code of Zoological Nomenclature, and hence the new names contained herein are available under that Code from the electronic edition of this article. This published work and the nomenclatural acts it contains have been registered in ZooBank, the online registration system for the ICZN. The ZooBank LSIDs (Life Science Identifiers) can be resolved and the associated information viewed through any standard web browser by appending the LSID to the prefix “http://zoobank.org/”. The LSID for this publication is: urn:lsid:zoobank.org:pub: 0A921D70–500F-4532-B507–248E0A589F29. The electronic edition of this work was published in a journal with an ISSN, and has been archived and is available from the following digital repositories: PubMed Central and LOCKSS.

## Results

### Systematic Paleontology

Mammalia Linneaus, 1785

Perissodactyla Owen, 1848

Tapiroidea Gill, 1872

Deperetellidae Radinsky, 1965

*Irenolophus* gen. nov.

urn:lsid:zoobank.org:act:5F545AE9–563D-4542-A14D-BAC6B9D201DD

### Type species

*Irenolophus qii* gen. et sp. nov.

### Included species

*Irenolophus primarius* (= *Teleolophus primarius*) (Qi, 1987)

### Horizon and locality

Arshanto Formation, early and middle Eocene, Huheboerhe area, Erlian Basin, Inner Mongolia, China.

### Etymology

*Iren*, after the Erlian (= Iren) Basin where the new cranial material found, meaning bright-colored in Mongolian; *loph*, crest, a commonly used root in early perissodactyl names.

### Diagnosis

Primitive, medium-sized deperetellid. Skull dolichocephalic with a shallow narial notch. Symphyseal region of the lower jaw relatively long and narrow. Dental formula: 3·1·4·3/3·1·4·3. P1 triangular in outline. P2–4 with a large lingual protocone and a relatively distinct paraconule, which is separated from the protocone by a shallow anterolingual groove (or confluent with the protocone). Upper molars with reduced and lingually depressed metacones. M3 outline trapezoid. p1 double-rooted. p2–3 with a low paraconid, and a weak, not bifurcated paralophid. p2–4 lacking an entoconid. Cristid obliqua of p3–4 joining the protolophid at the lingual side of the protoconid. m1–3 with reduced paralophid and cristid obliqua. Lunar with a relatively large contact with the magnum anteriorly. Mc V not highly reduced, as inferred from the corresponding facet on the unciform.

### Differential diagnosis

Differs from other deperetellids by a relatively shorter premolar series compared with molars, a trapezoid outline of M3 with a more distinct postmetacrista, p2–3 with a lower paraconid, p3–4 without a hypolophid and entoconid, and a more prominent paralophid and cristid obliqua on m1–3. Further differs from *Teleolophus* by a less distinct separation between the paraconule and the protocone on P2–4. Further differ from *Teleolophus* and *Deperetella* by a more distinct postmetacrista on M1–3, and p2–3 paralophid not bifurcated anteriorly. Further differs from *Deperetella*, *Diplolophodon*, and *Bahinolophus* by less molarized premolars. Further differs from *Diplolophodon* by a much larger size, and a double-rooted p1. Additionally, differs from *Bahinolophus* by a more lingually depressed metacone on M1–3 with a straighter protoloph and metaloph, a bulge at the posterobuccal corner of the crown on M1–2, and a double-rooted p1. Moreover, differs from *Pachylophus* by relatively sharper and slender transverse lophs on the upper and lower molars.

*Irenolophus qii* gen. et. sp. nov.

urn:lsid:zoobank.org:act:D93734DD-D264-4712-97D6-CE70D6B1AC4C

### Holotype

**Fig 1:**
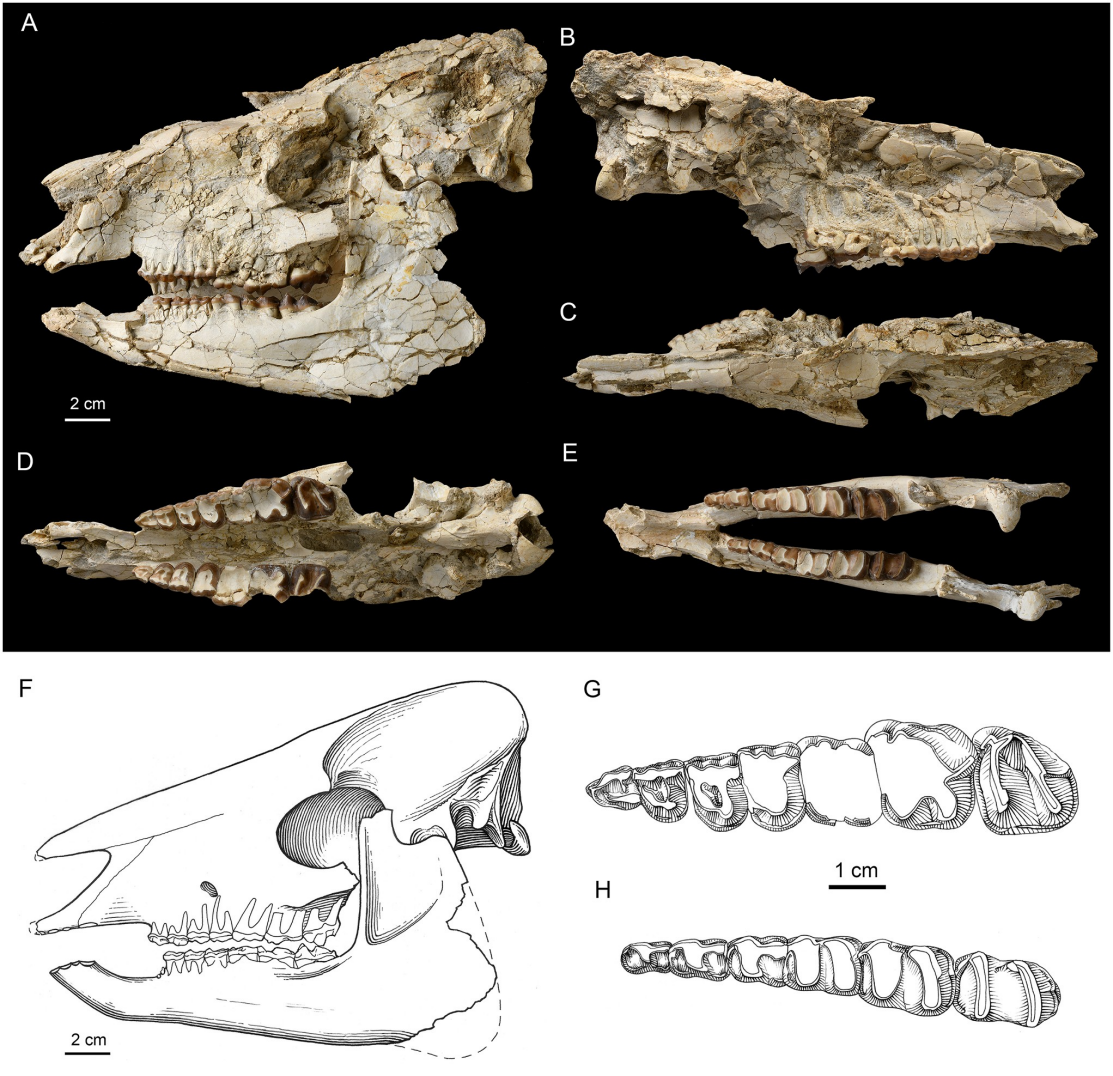
The skull and dentition of the primitive deperetellid tapiroid *Irenolophus qii* gen. et sp. nov. (IVPP V 25831) from the late early Eocene of the Erlian Basin, Inner Mongolia, China. (A) left lateral view of skull and mandible. (B) right lateral view of skull. (C) dorsal and (D) ventral views of the skull. (E) occlusal view of the mandible. (F-H) line drawing of the skull and mandible in lateral view (F), left P1-M3 in occlusal view (G), and right p2-m3 in occlusal view (H).

**Fig 2:**
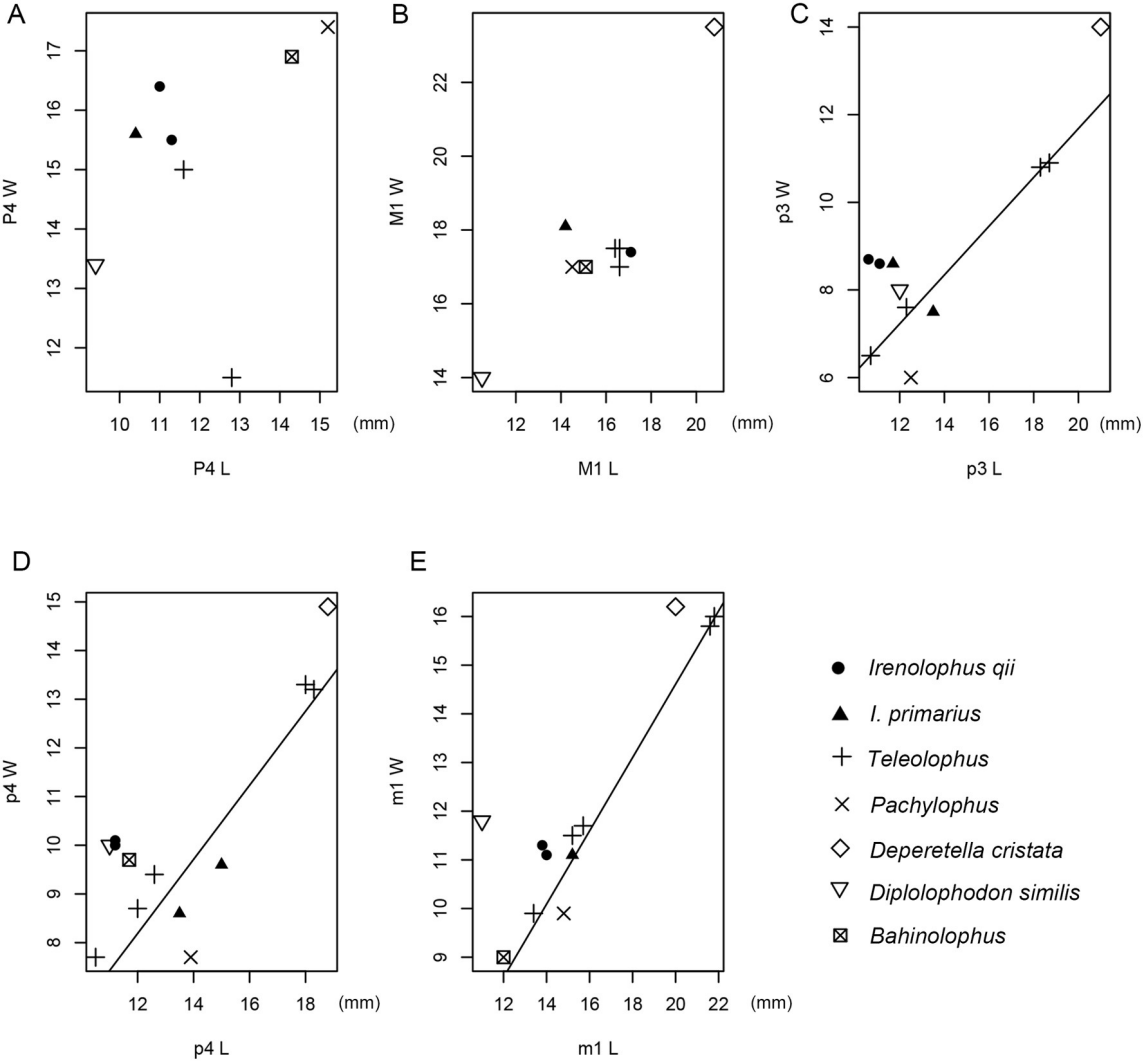
Scatter plot of P4-M1 (A-B) and p3-m1 (C-E) proportions in *Irenolophus* and other deperetellids, with regression lines for width as the function of length in *Teleolophus* (see S1 Table).

**Fig 3:**
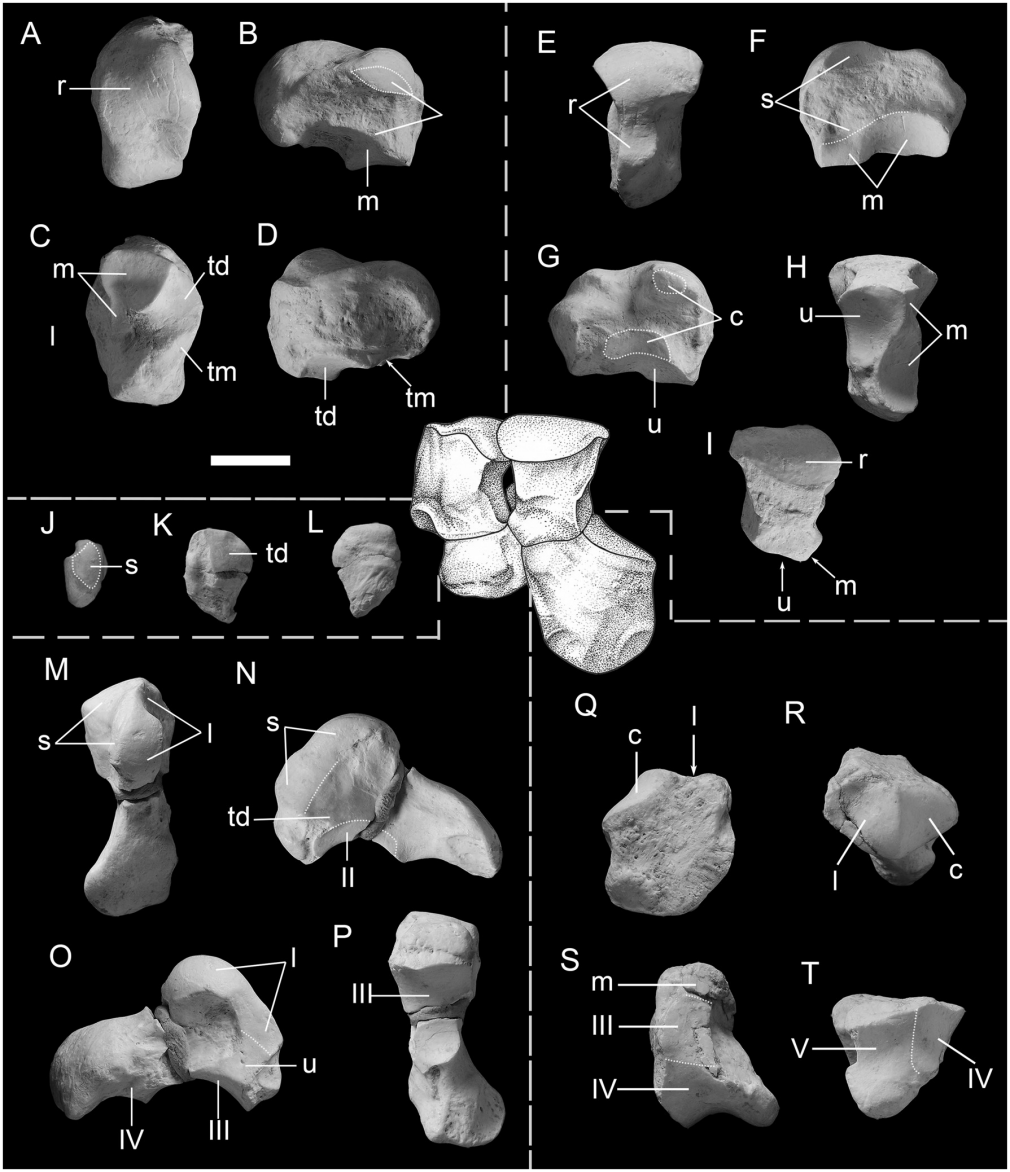
Carpals of the primitive deperetellid *Irenolophus qii* gen. et sp. nov. (IVPP V 25831). A reconstruction of articulated left carpals (reversed) in anterior view in the center. (A to D) right scaphoid in proximal (A), lateral (B), distal (C), and medial (D) views. (E to I) right lunar in proximal (E), medial (F), lateral (G), distal (H), and anterior (I) views. (J to L) right trapezium in proximal (J), anterior (K), and posterior (L) views. (M to P) right magnum in proximal (M), medial (N), lateral (O), and distal (P) views. (Q to T) right unciform in anterior (Q), proximal (R), medial (S), and distal (T) views. Abbreviations: c, cuneiform; l, lunar; m, magnum; r, radius; s, scaphoid; td, trapezoid; tm, trapezium; u, unciform; III, IV, V, third, fourth, and fifth metacarpals.

An associated skull, mandible, and some right carpals (scaphoid, lunar, magnum, and unciform) is housed at the IVPP, under the specimen number IVPP V 25831 (Figs 1–3, Tables 1–3).

**Table 1:** Measurements of skull and mandible of *Irenolophus qii* (mm).

	*Irenolophus qii* (IVPP V 25831)
**Skull**	
1. Basilar Length	225
2. Premaxilla-condyle, Length	241
3. Vertex Length	250^a^
4. Occipital condyle-postorbital process, Length	86
5. Premaxilla-postorbital process, Length	157
6. P1-condyle, Length	185
7. Postglenoid process-condyle, Distance	45
8. Nasal notch, Length	25^a^
9. Orbital-nasal notch, Distance	81^a^
10. Width at condyles	34
11. Width at post-tympanic process	47^a^
12. Width at zygomatic arch	−
13. Occiput Height	56^a^
14. Condyle, Height×Width	19×11
15. paracondylar-post tympanic process, Length	11
16. I1-M3, Length	145
17. Diastema I1-P1	54
18. P1-M3, Length	89
**Ratio %**	
12:1	−
12:2	−
4:5	54.8
7:6	24.3
17:18	60.5
**Mandible**	
1. Total Length	210^a^
2. p1-angular process, Length	60^a^
3. p1-m3 Length	88
4. Height at p2	28
5. Height at m1	32
6. Coronoid process, Height	123^a^
7. Condyle, Height	100^a^
**Ratio %**	
3:1	41.7
5:1	15.2
6:1	58.6
7:1	47.6

^a^ Approximate measurements.

**Table 2:** Measurements of upper teeth of *Irenolophus qii* and *I*. *primarius* (mm).

	*Irenolophus qii* (IVPP V 25831)		*I*. *primarius* (AMNH FM 81851)
Upper teeth	Left	Right	
I1 L	−	−	−
I1 W	−	−	−
I2 L	−	5.9^a^	−
I2 W	−	4.4	−
I3 L	7.6^a^	5.3	−
I3 W	3.6^a^	3.7	−
C L	6.5^a^	7.4	−
C W	3.9^a^	5.8	−
P1 L	9.6	9.7	−
P1 W	8.0	7.7	−
P2 L	9.7	9.5	8.7
P2 W	11.5	11.3	10.8
P3 L	9.9	10.2	9.7
P3 W	13.9	13.7	13.8
P4 L	11.0^a^	11.3	10.4
P4 W	16.4	15.5	15.6
M1 L	−	17.1^a^	14.2^a^
M1 W	−	17.4^a^	18.1^a^
M2 L	21.0	−	18.9
M2 W	20.3	−	−
M3 L	18.5	−	−
M3 W	19.7	−	−
P2–4 L	30.8	30.3	28.2
P1–4 L	39.9	39.9	−
M1–3L	49.9^a^	51.7^a^	−
**Ratios**			
P1-4/M1–3	79.9	77.1	−
P2-4/M1–3	61.7	58.5	−

^a^Approximate measurements.

**Table 3:** Measurements of lower teeth of *Irenolophus qii* and *I*. *primarius* (mm).

	*Irenolophus qii* (IVPP V 25831)		*I*. *primarius*	
Lower Teeth	Left	Right	AMNH FM 81799	IVPP V 5761^b^
i1 L	−	5.0	−	−
i1 W	−	5.3	−	−
i2 L	−	5.9	−	−
i2 W	−	4.5	−	−
i3 L	−	4.8	10.8	−
i3 W	−	3.4	5.1	−
c L	8.5	8.6	8.8	−
c W	4.9	−	5.4	−
p1 L	−	8.0^a^	9.9	−
p1 W	−	−	4.8	−
p2 L	9.7	9.8	11.3	10.9
p2 AW/PW	5.8/6.6	5.8/6.6	5.6/5.9	7.2
p3 L	10.6	11.1	13.5	11.7
p3 AW/PW	7.3/8.7	7.3/8.6	7.5/7.5	8.6
p4 L	11.2	11.2	13.5	15.0
p4 AW/PW	8.5/10.1	8.7/10.0	8.4/8.6	9.6
m1 L	13.8	14.0	−	15.2
m1 AW/PW	11.1/11.3	10.4/11.1	−	11.1
m2 L	17.4	17.8	19.2	17.6
m2 AW/PW	12.5/13.0	12.5/12.7	14.0/14.5	12.6
m3 L	19.3	19.7	20.5^a^	−
m3 AW/PW	12.7/12.5	12.6/12.3	15.9/15.7^a^	−
p2–4L	30.9	31.4	37.5	37.6
p1–4L	38.9^a^	39.4	47.3	−
m1–3L	49.3	50.2	57.8^a^	−
**Ratios**				
p1-4/m1–3	78.9	78.4	81.9^a^	−
p2-4/m1–3	62.7	62.4	64.9^a^	−

^a^Approximate measurements.

^b^Measurements from the table 13 of Qi [14].

### Horizon and locality

Base of the Arshanto Formation, late early Eocene, Nuhetingboerhe, Erlian Basin, Inner Mongolia, China.

### Etymology

The specific name dedicated to Prof. Tao Qi, for his contributions to research and fieldwork in the Erlian Basin.

### Differential diagnosis

Differs from *Irenolophus primarius* in having relatively shorter premolar series compared with the molars (a ratio of p1–4 to m1–3 length is about 0.78), P2–4 paraconule more distinct and separated from the protocone by a shallow anterolingual groove, p2–4 relatively shorter and wider with a wider talonid, a weaker rib-like crest on the lingual side of the hypoconid, p3-p4 cristid obliqua more buccally extended towards the protolophid, and m1–2 paralophid and cristid obliqua slightly less reduced.

### Comparative description

#### Skull

The skull is laterally compressed, and the right side is much more damaged and weathered than the left (Fig 1A and 1B, Tables 1). The skull is dolichocephalic with the facial length (from postorbital process of the frontal to the anterior tip of the premaxilla = 140 mm) is ~27% longer than the length of the cranium (110 mm).

The body of the premaxilla preserves three alveoli for the incisors. Although the alveolus of I1 is partly damaged, it appears to be as large as the rounded alveolus of I2, which is in turn slightly larger than the rounded alveolus of I3. The ascending process of the premaxilla contacts the nasal proximally and maxilla posteriorly as in *Heptodon*. However, the premaxilla does not contact the nasal in *Colodon* [8] and *Lophialetes* [9]. The narial notch is situated over the postcanine diastema as in *Heptodon*, and closer to the canine than to P1. By contrast, the narial notch is retracted to the level of M2 in *Lophialetes* [9], and the anterior orbit in *Colodon* [8]. The maxilla is broad and slightly depressed anterior to the infraorbital foramen; the latter is located dorsal to the P3 and P4 boundary as in *Colodon*, but in a relatively lower position compared with those of *Heptodon*, *Colodon*, and *Lophialetes*. In *Colodon*, a distinctive trough is present on the ascending process of the maxilla, and curls onto the posterodorsal nasal [8]. The maxilla bears a relatively large, oval-shaped canine alveolus, which is separated from I3 by a short diastema as in *Heptodon* and *Lophialetes*. The postcanine diastema is long (about 24 mm). The roots of the cheek teeth are interesting in two aspects. One aspect is that the roots are largely exposed on the lateral side with even some tips visible. This morphology is in part because of weathering, but the probability that the buccal roots are actually very close to the lateral border of the maxilla can not be excluded. The second aspect is that the roots of the premolars are nearly vertical, while those of the molars are inclined considerably posteriorly. The nasal is long and its broken anterior tip overhangs the I3 as in *Heptodon*. By contrast, the nasal is short and retracted in *Colodon* and *Lophialetes*. The sutures between the nasal and frontal, lacrimal, and maxilla are almost completely obliterated because of ossification or damage. The exception is a visible suture anterodorsal to the orbit, showing an extensive contact between the frontal and lacrimal. The frontal is flat, but the postorbital process of the frontal is broken away. The frontal ridges converge posteriorly to a sharp, long sagittal ridge at the level of the point slightly anterior to the postglenoid fossa (Fig 1C).

The orbit is moderately large and rounded as in *Heptodon*, but relatively smaller than that of *Lophialetes*. An oval lacrimal foramen is present along the anterior border of the orbit. Although the zygomatic arch is damaged, a partial jugal is preserved below the orbit, and its ventral border forms a sharp ridge as in other compared taxa. In ventral view (Fig 1D), the posterior border of the palate is between the posterior border of the M2s, as in *Heptodon*. The pterygoid process of the alisphenoid extends anteroventrally with an oval posterior opening of the alisphenoid canal situated posterodorsally. The glenoid fossa is partially damaged, but the preserved part is flat. The postglenoid process is flat, and strong as in *Heptodon*, and oriented anterolaterally with a postglenoid foramen. On the medial side of the glenoid fossa, there is an oval foramen separated from the middle lacerate foramen. The external acoustic meatus is open on the ventral side, and the posttympanic process seems more slender and less ventrally extended than the postglenoid process. The paracondylar process (better preserved on the right side) is laterally compressed, and more ventrally extended than the postglenoid process as in *Heptodon*, but does not extend beyond the ventral border of the occipital condyles. The mastoid process was exposed on the lateral side, separating posttympanic process and paracondylar process with a small mastoid foramen placed near the divergence of the nuchal crest.

The occipital has been heavily deformed and damaged, so that the outline of the occipital is not discernable. The occipital condyles are widely separated dorsally and closely spaced ventrally. The lateroventral border of the condyle (linea divisa condyli) is a somewhat blunt ridge, and forms the angle of about 55° with the long axis of the skull. The suture between the basiocciptial and basisphenoid is obliterated, but is probably near the muscular tubercles. The basioccipital bears a blunt, pinched median ridge anteriorly. The left petrosal is intact, but is mostly covered by matrix.

#### Lower jaw

The lower jaw is laterally compressed like the skull, with the right mandible positioned higher than the left one (Fig 1A and 1F). The angular and coronoid processes are broken off. The body of the lower jaw has three pairs of incisors. Based on the incisor alveoli, it is reasonable to infer that i1 with a rounded alveolus is slightly larger than i2, and i3 is the smallest incisor. An oval, relatively large canine alveolus is closely appressed to that of i3, and is larger than those of the incisors. The symphyseal region is relatively long and narrow with a posterior border situated at the level of the anterior border of p1 (Fig 1E). The dorsal surface of the symphyseal region is transversely deeply concave. The symphyseal region rises anteriorly as in *Heptodon*, although it seems more procumbent on the left side because of the deformation (Fig 1A). The horizontal ramus is slender and increases in height slightly toward the posterior side with a straight ventral border as in *Heptodon*. In contrast to the roots of upper cheek teeth, the roots of the lower check teeth are more exposed on the lingual side than the buccal one, are more distinctly exposed on p2-m1 than on m2–3, and are oriented nearly vertically. The mandibular foramen is situated at the level of the alveolar border. The high condylar process is partly damaged, but the articular surface is slightly convex anteroposteriorly, laterally flat, and curves to the posteromedial side of the condylar process. The tip of the coronoid process has been broken off, and the mandibular incision is open and wide. The coronoid crest is nearly vertical, and the condyloid crest slants anterodorsally.

#### Teeth

P1 is triangular in outline with a wider posterior border (Fig 1D and 1G, Table 2), in contrast to the oval outline in *Teleolophus*. The parastyle is prominent, large and distinctly separated from the paracone. The metacone is much smaller than the paracone with a slightly convex buccal surface. The protoloph is long, posterolingually extended, and joins the anterolingual base of the paracone. The metaloph (or metaconule) is rather short and slender, rises to the ectoloph, and is not in contact with the protoloph. A weak, indistinct groove is discernible in the middle of the lingual side of the protoloph, demarcating the separation of the paraconule and the protocone. P2 is roughly rectangular in outline with a curved anterolingual border, and its width is slightly greater than its length. The paracone has a convex buccal side, the metacone is close to the paracone (slightly convex on the buccal side), and has nearly merged with the ectoloph. The protoloph is posterolingually curved from the anterolingual base of the paracone to the large protocone. The metaloph, which is as strong as the protoloph, is slightly arched posteriorly, nearly parallel to the posterior border of the tooth, and joins the ectoloph in a high position. On the anterolingual side of the protocone, there is an indistinct groove for the incipient separation of the paraconule and the protocone as in P1. In *Teleolophus*, the separation between the paraconule and the protocone is much more distinct. P3 is roughly rectangular in outline with a rounded lingual border. The parastyle is larger than that of P2, and the metacone has a slightly less convex buccal side than the paracone. The protoloph is posterolingually curved, enlarged in the middle (representing the paraconule), and constricted on the either side. The protoloph joins the protocone by a narrow ridge that is a short distance to the lingual end of the protocone, as in *Teleolophus*. On the anterolingual side of the protocone, there is a distinct groove, which is more distinct than that on P2, separating the paraconule from the protocone. P4 is relatively shorter and wider than P3 (Fig 2A), but its detailed morphology is obliterated by the heavy wear. The cingula of P2–4 are complete and distinct on the buccal and lingual borders. By contrast, the protoloph and metaloph of P2–4 form a complete loop in *Lophialetes*, and are separated completely in *Colodon*. The P2 metaloph is absent, and the metaloph of P3–4 is lower and weaker than the protoloph in *Heptodon*.

M1 is too heavily worn to show any morphological details (Fig 1D and 1G; Table 2). The outline of M1 is roughly square and as long as wide (Fig 2B). M2 is as long as wide, and also heavily worn with most features indiscernible. The paracone is broadly convex on the buccal side, and the metacone is strongly depressed lingually and concave as in *Teleolophus* and *Colodon*. However, the metacone of M1–2 is flat or slightly convex, and relatively more buccally positioned in *Heptodon*. The postmetacrista is short and posterobuccally extended as in *Teleolophus* and *Colodon*, but the upper molars of *Lophialetes* are characterized by long postmetacrista. The parastyle is strong, and mainly anterior to the paracone. At the posterobuccal corner of the tooth, there is a bulge at the base as in *Teleolophus*, and a distinct cingulum is present at the posterobuccal base in *Heptodon* and *Colodon*. The cingulum is weak on the posterior side and interrupted on the lingual sides of the protocone and hypocone. M3 is slightly worn and roughly trapezoidal in outline, but the M3 of *Teleolophus* is roughly square. The parastyle is large, anterobuccal to the paracone, and separated from the latter by a distinct groove on the buccal side. The paracone is broadly convex on the buccal side and tilts posteriorly. The posterior loph is composed of the nearly confluent centrocrista and metaloph, and their boundary is marked by a strongly lingually depressed, flat, and reduced metacone that is placed midway between the paracone and hypocone as in *Colodon*. The postmetacrista is much more reduced and shorter than that of M2, but is still distinct. The M3 centrocrista and metaloph are completely confluent, and the metacone is absent in *Teleolophus*. The cingulum is complete around the anterior, lingual, and posterior borders, but is nearly absent on the buccal side.

The crown of p1 is broken off and only two roots are preserved (Fig 1E). The p2 is rectangular in outline and anteroposteriorly elongated with a talonid wider than the trigonid (Fig 1E and 1H; Table 3). The paraconid is weak and anterobuccally placed, bearing a tiny cristid on the lingual side. By contrast, the p2 paralophid is longer and more arched in *Lophialetes*. The protolophid is short and the metaconid is posterolingually placed relative to the protoconid. The talonid is relatively wide without the entoconid. The cristid obliqua is straight, extending anteriorly from the large hypoconid to the protoconid. The cingulids are prominent on the buccal side, but very weak on the lingual side of the talonid. The cingulid on the lingual side of the trigonid is distinct and continuous with the anterior cingulid, which rises to join the paraconid. The p3 is moderately worn. It has a wider protolophid than in p2, and the talonid is prominently wider than the trigonid as in *Colodon* (Fig 2C). In *Teleolophus*, the talonid is nearly as wide as the trigonid, and the paralophid is somewhat bifurcated. The lingual side of the hypoconid has a small projection. The entoconid is absent except for a very weak cingulid on the lingual side of the talonid, but the entoconids are more prominent in *Teleolophus*, *Colodon*, and *Lophialetes*. The p4 is similar to p3, except that the paraconid is nearly absent, the trigonid is relatively shorter and wider, and the talonid is relatively wider compared with the trigonid as in *Colodon* (Fig 2D). A weak cingulid at the lingual base of the talonid represents the incipient entoconid, which is more prominent in *Teleolophus*, *Colodon*, and *Lophialetes*. The hypoconid bears a more distinct projection on the lingual side than in p3.

The m1 is heavily worn, and the m2 is moderately worn and slightly larger (Figs 1E, 1H and 2E; Table 3). The protolophid is parallel with the hypolophid, and both of them are slightly posterolingually oblique. The paralophid and the cristid obliqua are very reduced as in *Teleolophus* and *Colodon*, they are less reduced in *Heptodon*, and they are strong and long in *Lophialetes*. The trigonid is short, but the talonid is longer and slightly wider. The cingulid is completely absent on the lingual side, faint at the base of the ectoflexid, and distinct on the posterior side. The m3 is slightly worn and generally similar to m1–2 except for the talonid being slightly narrower than the trigonid, and having a more convex posterior border with a highly reduced hypoconulid as in *Teleolophus* and *Colodon*. The cingulid is nearly absent on the buccal side. By contrast, the m3 hypoconulid is enlarged into a third lobe in *Lophialetes*, and is short and narrow in *Heptodon*.

#### Carpals

Five right carpals, including the scaphoid, lunar, trapezium, broken magnum, and unciform, are preserved, and all are probably from the same individual (Fig 3, Table 4).

**Table 4:** Measurements of some carpals of *Irenolophus qii* (IVPP V 25831) (mm) Measurements correspond to the tables 14, 15, 17, and 42 of Qiu and Wang [15].

	Scaphoid	Lunar	Magnum	Unciform
1.	15.4	16.2	10.5	18.7
2.	14.9	10.5	19.0^a^	17.9
3.	10.6	14.0	11.6	19.4
4.	21.8	21.1	30.0^a^	10.0
5.	13.3×16.5	2.8	7.0×12.7^a^	11.0
6.	9.5×14.5	9.7	8.9×17.0	10.1
7.	8.8×11.6	50^a^	10.4×15.0^a^	10.5
8.	−	−	−	7.8
9.	−	−	−	10.7
**Ratio (%)**				
1.	103.9	64.8	55.0	96.5
2.	70.6	66.5	89.8	92.3
3.	68.0	346.2	163.2	90.5
4.	−	−	38.8^a^	96.4
5.	−	−	34.8	72.4
6.	−	−	55.4	−

^a^ Approximate measurements.

On the proximal side, the radial facet of the scaphoid is roughly oval in outline with a strong convexity at the anterolateral part (Fig 3A–3D). The posterior process is relatively wide and flat, extending smoothly from the radial facet to the distal side as in *Deperetella*. On the lateral side, there are two lunar facets that tend to converge on the posterior side (Fig 3B) as in *Heptodon* and *Deperetella*. A proximal one of the lunar facets is wide and short with a podiform outline, whereas the distal one is narrow and more posteriorly extended. On the distal side, there are three facets for the magnum, trapezoid, and trapezium (Fig 3C) as in *Deperetella*. The magnum facet is nearly flat on the anterior half, and deeply concave on the posterolateral part, but the facet is concave and elongated in *Lophialete*s. The magnum facet extends posteriorly along the distal lunar facet as a strip-like, flat facet. The trapezoid facet is slightly saddle-shaped, and separated from the magnum and trapezium facets by a sharp and weak ridge, respectively. The trapezoid facet is concave and relatively more posteriorly placed in *Colodon*. The trapezium facet is small, slightly concave, and mostly directed distally, and the trapezium facet is absent in *Heptodon*, *Colodon*, and *Lophialetes*.

The lunar is relatively narrow and long in proximal view (Fig 3E). The anterior radial facet is convex anteroposteriorly, and extends considerably onto the anterior surface (Fig 3I) as in *Teleolophus* and *Deperetella*. The posterior radial facet is narrow, pointed at the posterior end, slightly concave, and placed medially as in *Teleolophus* and *Deperetella*. The facet is in the middle in *Lophialetes* and much reduced in *Colodon*. The medial border of the radial facet is slightly concave. On the medial side, there are two scaphoid facets (Fig 3F): the proximal one is prominent, slightly concave, mainly confined to the anterior half; and the distal one is narrow, elongated, and confluent with the magnum facet on the anterior part. Laterally, there are two flat facets for the cuneiform (Fig 3G): the proximal one is small and oval; and the distal one is larger, more posteriorly extended, and roughly lunate in shape. On the distal side, the unciform facet occupies the anterolateral portion (Fig 3H) and is directed slightly distolaterally with a concave facet. The magnum facet is composed of two parts (Fig 3F and 3H) as in *Teleolophus*, *Heptodon*, and *Colodon*, but the anterior magnum facet is absent in *Lophialetes* and *Deperetella*. The anterior one, which is confluent with the distal scaphoid facet, is distinct, flat, and mainly vertically placed, but inclined somewhat medially. The posterior magnum facet is spherically concave, considerably elongated anteroposteriorly, and faces medially more than distally.

The trapezium is flat and irregular in outline (Fig 3J–3L) as in *Heptodon*, and the trapezium is rod-like or has a posterior process in *Lophialetes* and *Deperetella*, respectively. The trapezium bears a small scaphoid facet proximally and a large trapezoid facet on the lateral side as in *Deperetella*. However, the scaphoid facet is absent in *Lophialetes*. The two facets are separated by a distinct ridge. The distal end of the trapezium is pointed. Measurements (in mm): Width, 8.7; Length, 5.6; Height, 11.8.

The magnum was broken into anterior and posterior parts, and some fragments are missing between them (Fig 3M–3P). The magnum has a roughly square anterior surface with a convex distal border, a relatively high hump, and a posterior process transversely expanded at the posterior end as in *Teleolophus*, *Deperetella*, and *Lophialetes*. By contrast, the hump of the magnum is relatively more posteriorly situated and the posterior process is more distally extended or even medially projected in *Heptodon* and *Colodon*. The anterior scaphoid facet is slightly anteromedially inclined, and nearly flat, but the posterior one is on the medial side of the hump, vertically placed, and flat (Fig 3M and 3N). By contrast, the anterior scaphoid facet is anteroposteriorly convex in *Lophialetes*. The lunar facet is composed of two parts: the anterior one is flat, nearly vertical, and confluent with the unciform facet; and the posterior one is situated on the lateral side of the hump, and is somewhat spherically convex (Fig 3M and 3O) as in *Teleolophus*, *Heptodon*, and *Colodon*. The anterior lunar facet is absent in *Lophialetes* and *Deperetella*. On the medial side, distal to the flat trapezoid facet, the Mc II facet is band-like, flat, and nearly vertically placed, although the bone was broken in the middle (Fig 3N). Although the Mc III facet is not complete, it is moderately concave anteroposteriorly and slightly convex laterally on the distal end (Fig 3P). The Mc III facet is more concave in *Teleolophus*, *Deperetella*, and *Lophialetes*. Posterolateral to the Mc III facet, there is a depression probably for the Mc IV on the lateral side of the posterior process (Fig 3O) as in *Teleolophus* and *Lophialetes*, but the Mc IV facet is absent in *Heptodon*, *Colodon*, and *Deperetella*.

The unciform is nearly as high as wide, and has a short posterior process (Fig 3Q–3T) as in *Heptodon*. The unciform is relatively higher in *Deperetella* and *Colodon*, and relatively lower with a longer posterior process in *Lophialetes*. The proximal surface is divided into two parts by an elevated, sagittal ridge (Fig 3R). The lunar facet is roughly trapezoidal in outline, concave at the anteromedial portion, and convex in its posterolateral part. The cuneiform facet is triangular, slightly convex anteroposteriorly and concave laterally. On the medial side, the magnum and Mc III facets are confluent and flat with the latter higher than the former. The medial articular facet curves gently to the distal surface, which bears confluent Mc IV and Mc V facets (Fig 3S and 3T). The Mc IV facet is triangular and slightly saddle-shaped (concave anteroposteriorly and slightly convex laterally), and the Mc V facet is quadrilateral in outline (concave anteroposteriorly and nearly flat transversely). The Mc V facet is relatively large, indicating that the Mc V is relatively strong and not highly reduced (Fig 3T), as in *Heptodon*. The Mc V fact is more reduced in *Deperetella*, *Lophialetes*, and *Colodon*.

#### Comparisons

*Irenolophus qii* represents a basal Asian endemic deperetellid and shows following characters shared with deperetellids for the upper check teeth (Fig 1D and 1G): premolar series is elongated relative to molars (in particular for P1 which is longer than wide); the upper premolars have convex paracones and nearly flat metacones, with the latter merged with the ectolophs; the protolophs of P3-P4 join the metalophs a short distance before the lingual ends of the protocones; the grooves are present at the anterolingual sides of the protocones demarcating the separation of the paraconules; M2 has a bulge on the posterobuccal side; and the cingula of upper premolars are nearly complete on the lingual sides. For the lower check teeth (Fig 1E and 1H): the premolar series are relatively elongated compared with molars; p1 has two roots; p2 and p3 are longitudinally elongated; m1-m2 are bilophodont with parallel and slightly oblique protolophids and hypolophids; and m3 lacks an enlarged hypoconulid. The manus of *Irenolophus* (Fig 3) also is similar to that of *Teleolophus* and *Deperetella* in having the posterior process of the scaphoid relatively wide and flat, the lunar relatively long and narrow with a slightly concave medial border of the radial facet, the trapezium flat with a scaphoid facet, the magnum with a relatively high, anteroposteriorly short hump, and an expanded posterior process.

On the other hand, *Irenolophus* is more primitive than *Teleolophus* and *Deperetella* in having relatively shorter premolar series compared to the molars, P1 triangular in outline with the protoloph joining the protocone, the P2-P4 with less divided protolophs and metalophs on the lingual sides, the upper molars with more distinct metacones and postmetacristae, the lower premolars less molariform without entoconids, p2-p4 with wider talonids compared with the trigonids, p2–3 paralophid not bifurcated anteriorly with lower paraconid, and lower molars with less reduced paralophids and cristids obliquae. Furthermore, the manus of *Irenolophus* (Fig 3) is more primitive than that of *Teleolophus* [6] and *Deperetella* [1] in having the scaphoid with a relatively longer, narrower distal lunar facet and a posterior extension of the posterior magnum facet, the lunar with a distinct anterior magnum facet and a relatively longer and narrower distal scaphoid facet, the magnum with a relatively large anterior lunar facet and a less concave Mc III facet, and an unciform with a shorter posterior process, and a relatively larger Mc V facet more distally directed. The main differences between *Irenolophus* and other deperetellids are listed in the differential diagnosis.

*Irenolophus primarius* (= *Teleolophus primarius*) (Qi, 1987)

#### Holotype

A left mandible with p2-m2, IVPP V 5761.

#### Referred specimens

IVPP V 5762, a left mandible with m2–3; IVPP V 5763, associated teeth, several carpals, and phalanges; IVPP V 5764, two m1s; IVPP V 5765, two m1s; AMNH FM 81851, a left maxilla with P2-M1, and M2–3 fragments; AMNH FM 81799, a lower jaw with right c, left i3, c, and p1-m3; AMNH FM 81850, a right mandible with dp3–4, and m1; AMNH FM 81800, a right mandible with dp3–4.

#### Horizon and locality

Arshanto Formation, late early Eocene to early middle Eocene, Wulanboerhe and Huheboerhe localities.

#### Differential diagnosis

Differs from *Irenolophus qii* in having relatively longer premolar series compared with the molars (a ratio of p1–4 to m1–3 length is about 0.82), the protoloph and metaloph of P2–4 forming a nearly continuous loop, p2–4 relatively longer and narrower with a talonid as wide as the trigonid, and a more distinct rib-like crest on the lingual side of the hypoconid, p3-p4 cristid obliqua more lingually extended towards the protolophid, and m1–2 paralophid and cristid obliqua slightly more reduced.

#### Comments

Qi [14] described two species of *Teleolophus*, ‘*T*.’ *primarius* and ‘*T*.’ ? *rectus*, from the Arshanto Formation in Huheboerhe area. ‘*Teleolophus*’ *primarius* is distributed from the base to the upper parts of the Arshanto Formation at Wulanboerhe and Huheboerhe. Compared with *Teleolophus medius*, ‘*T*.’ *primarius* has p2–3 paralophid not bifurcated anteriorly, the entoconid absent on p2–4, and a less reduced paralophid on m1–3. Those features are in turn very similar to those of *Irenolophus*, suggesting that ‘*Teleolophus*’ *primarius* is more reasonably included in *Irenolophus* than in *Teleolophus*.

Qi [14] named another species ‘*Teleolophus*’ ? *rectus*, which is only known from two fragmentary mandibles with p4-m1 (IVPP V 5766), from the base of the Arshanto Formation at Wulanboerhe. However, the diagnosis of the species includes “p4 paralophid closer to lingual edge of crown, cristid obliqua (= metalophid) aligned almost with the medial part of the talonid; entoconid indistinct” [14], which are not sufficient to separate it from other species of *Teleolophus* or *Irenolophus*. Furthermore, the holotype is too fragmentary, and we treat the species ‘*Teleolophus*’ ? *rectus* as a *nomen dubium*.

**Fig 4:**
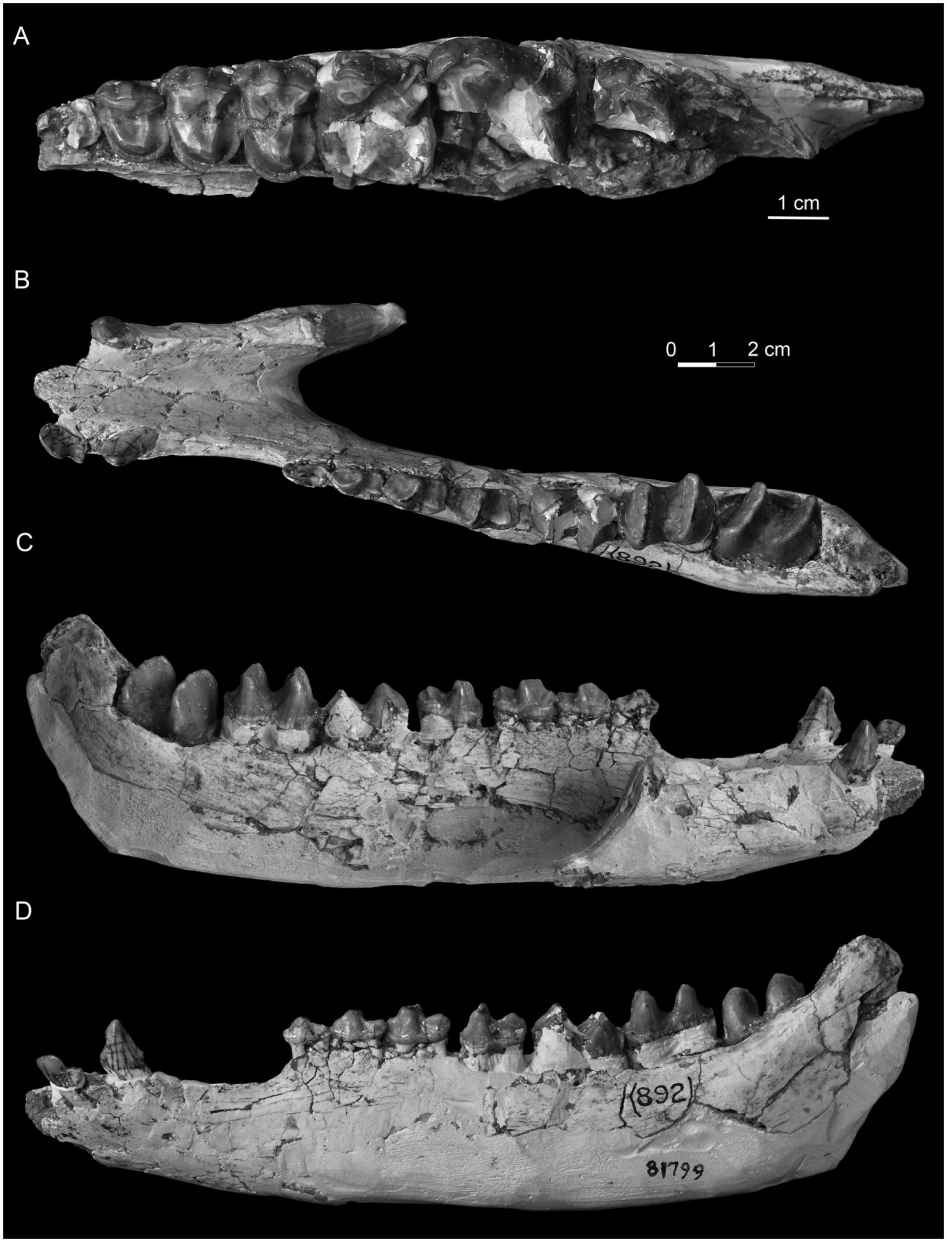
Upper cheek teeth and lower jaw of deperetellid *Irenolophus primarius* from the Arshanto Formation at Huheboerhe of the Erlian Basin, Inner Mongolia, China. (A) occlusal view of the left maxilla with P2-M1, and M2–3 fragments (AMNH FM 81851). (B-D) lower jaw with right c, left i3, c, and p1-m3 in occlusal (B), lingual (C), and buccal (D) views (AMNH FM 81799).

Radinsky [1] mentioned some specimens of *Teleolophus* from Camp Margetts area collected by CAE (the Central Asiatic Expedition of AMNH in 1920s), and assigned them to *Teleolophus* cf. *T*. *medius*. He further considered all of them to have come from the “Irdin Manha” beds [1]. Recent investigations in the Erlian Basin clarified that the so-called “Irdin Manha” beds and “Houldjin gravels” are actually equal to “Arshanto Formation” and “Irdin Mahan Formation” in the Camp Margetts area [16–19]. Thus, the specimens AMNH FM 81851 (Fig 4A), 81799 (Fig 4B–4D), 81850, and 81800 from the “Irdin Manha” beds, 7 miles southwest of Camp Margetts are from the Arshanto Formation at Huheboerhe. The specimen AMNH FM 81853, recorded as from ?“Irdin Manha” at 10 miles southwest of Camp Margetts, is from upper portion of the Arshanto Formation, Changanboerhe. The other two specimens (AMNH 81854, 81855) from the same locality, recorded as from ?“Houdjin gravels,” actually came from the Irdin Manha Formation. However, Radinsky [1] considered both of the specimens as being from the same horizon as the fossils from 7 miles southwest of Camp Margetts. The specimen AMNH FM 81798 from the “Irdin Manha” beds, 5 miles east of Camp Margetts (Daoteying Obo), is probably actually from the Irdin Manha Formation based on its preserved condition.

The materials of ‘*Teleolophus*’ from the Arshanto Formation, 7 miles southwest of Camp Margetts (Huheboerhe) are all recorded with the field number 892, and thus are probably from the same locality and represent the same species. As pointed out by Radinsky [1], they differ from *T*. *medius* in having “P2–4 relatively shorter and wider, M2 metacone less reduced, and p3-m3 paralophids slightly less reduced” (Figs 2 and 4). Furthermore, the CAE material from Huheboerhe is different from *Irenolophus qii* in having P3-P4 protoloph and metaloph almost confluent on the lingual side (Fig 4A), p3 paraconid more distinct, and p3-p4 cristid obliqua slightly more lingually extended towards the protolophid with a rib-like crest on the lingual side of the hypoconid (Fig 4B–4D). Those features are very similar to *Irenolophus primarius* except for the upper dentitions, which are unknown for *I*. *primarius*. Thus, we assign these specimens of *Teleolophus* cf. *medius* from Huheboerhe in the Arshanto Formation to *Irenolophus primarius*. Other CAE specimens assigned to *Teleolophus* cf. *T*. *medius* by Radinsky [1] will be treated separately in the future studies.

### *Irenolophus* sp

#### Material

A right mandible with fragmentary p3-m2 and complete m3 (IVPP V 25832).

#### Locality and horizon

Base of the Arshanto Formation at Nuhetingboerhe, late early Eocene, Erlian Basin, Inner Mongolia, China.

#### Comparative description

**Fig 5:**
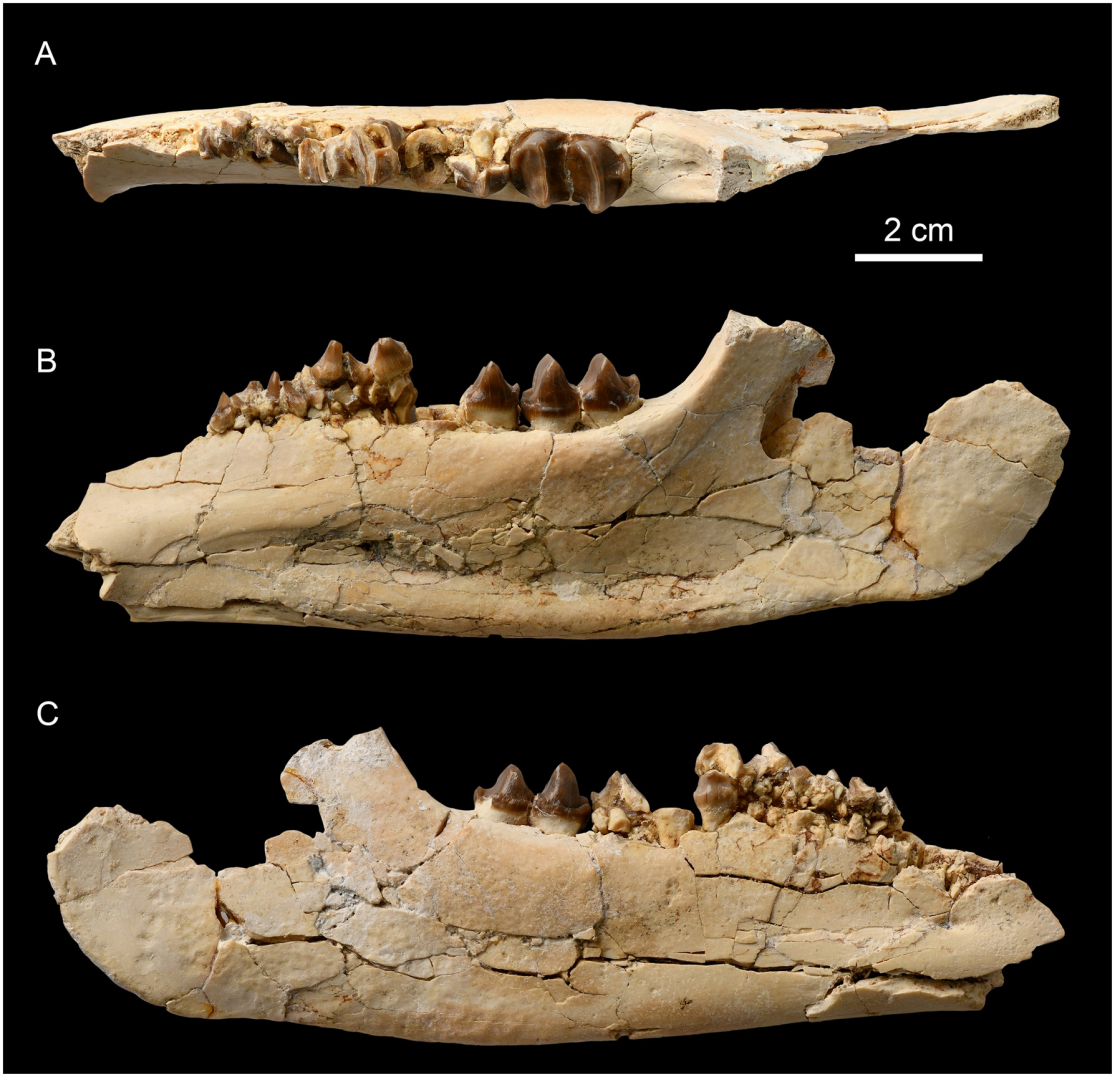
Right mandible of deperetellid *Irenolophus* sp. (IVPP V 25832) from the Arshanto Formation at Nuhetingboerhe of the Erlian Basin, Inner Mongolia, China. (A) occlusal, (B) lingual, and (C) buccal views of the right mandible.

The m3 of IVPP V 25832 is generally similar to that of IVPP V 25831 in morphology and size, but the former has a slightly higher crown that is steeper on the anterior surfaces of the hypolophid and the protolophid, has a weak cingulid on the buccal side, and has a slightly more distinct hypoconulid (Fig 5A–5C). Moreover, the mandibular foramen of IVPP V 25832 is located below the alveolar border (Fig 5B), and that of *Irenolophus qii* is relatively smaller and more posteriorly and dorsally placed. Considering that only m3 of IVPP V 25832 is complete and other teeth are fragmentary, we consider the specimen as *Irenolophus* sp.

#### Enamel microstructure

The Hunter-Schreger Bands (HSB) configurations of perissodactyls consist of four types: transverse, curved, compound, and vertical [5, 20]. The tooth enamel of deperetellids is conventionally characterized as vertical HSB, which was considered previously to be also present in rhinocerotoids, astrapotheres, and pyrotheres [21–23]. However, von Koenigswald et al. [5] suggested that the cheek tooth enamel of deperetellids as having a compound HSB with transverse HSB in an inner zone and vertical HSB in an outer zone, similar to that of *Hyrachyus* and *Uintaceras*. The vertical HSB, present in rhinocerotoids is probably derived from a compound HSB [5]. This hypothesis of a vertical HSB origin is different from two other hypotheses suggesting that it evolved from a curved HSB [22] or appeared as a new structure from no decussation prisms [24].

**Fig 6:**
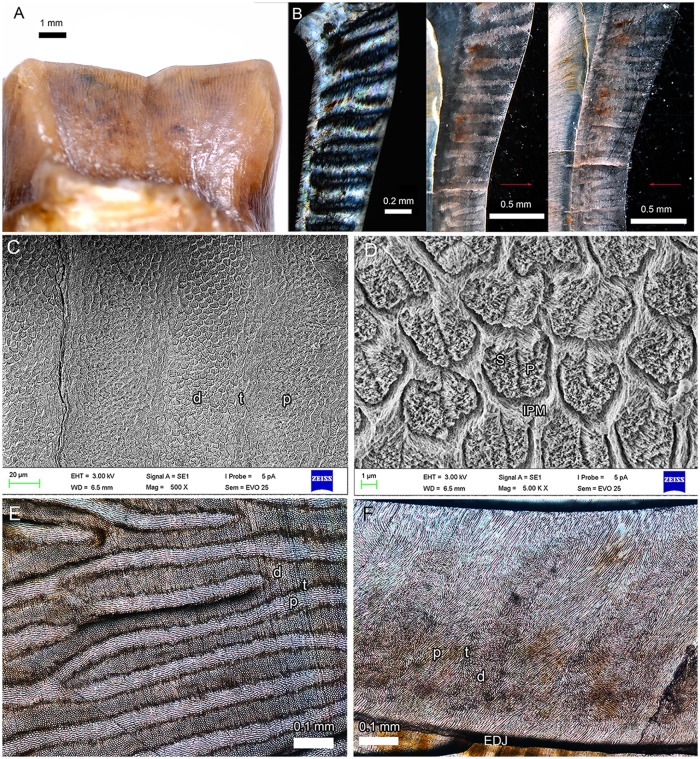
Enamel microstructure of the second lower molar of deperetellid *Irenolophus* sp. (IVPP V 25832). (A) vertical Hunter-Schreger Bands (HSB) in anterior view of the protolophid. (B) vertical HSB in the cross sections with polarized light and ordinary light; arrows showing the ordinary light directions. (C) vertical HSB and transitional zones in cross section. (D) prism shape in cross section. (E) parazone, diazone, and transitional zones in the tangential section. (F) Schmelzmuster in the vertical section. Abbreviations: d, diazone; EDJ, enamel-dentine junction; IPM, interprismatic matrix; P, prism; p, parazone; S, seam; and t, transitional zone.

The cross section of lower molar enamel in *Irenolophus* sp. (IVPP V 25832) shows a vertical HSB, which is clearer in polarized light (Fig 6A and 6B). Each diazone and parazone are about 8–9 prisms in width, and separated by a narrow, elongated transitional zone (Fig 6C). In the cross section, the shapes of the prisms vary from rounded to oval. The prism sheaths are usually open toward the outer enamel surface (OES), and the prisms are arranged in alternating positions (Fig 6D). The diameter of the prism head is about 4 μm. The interprismatic matrix (IPM) surrounds the prism with the crystallites forming an angle of less than 90 degrees with those of the prism. Some prisms have irregular seams, which usually run across the prism in its middle (Fig 6D). In the tangential section, the bands are bifurcate (Fig 6E). The parazone prisms are relatively elongated and bright, but the diazone prisms are rounded and dark. The transitional zone between them is narrow and the darkest.

The Schmelzmuster of *Irenolophus* in the vertical section shows that the enamel is about 0.6 mm thick (Fig 6F). The Schmelzmuster is mainly composed of HSB, and the parazone is wider than the diazone with a narrow transition zone between them. The inclination of HSB to the enamel-dentine junction (EDJ) is about 20°. Adjacent to the EDJ, there is a narrow layer (about 50μm) of radial enamel, which is either parallel or somewhat continuous with the prisms of the parazones. A similar narrow layer of radial enamel adjacent to outer enamel surface (OES) also is discernable.

To sum up, the cheek tooth enamel of *Irenolophus* only has vertical HSB, which probably originated from curved HSB such as present in *Heptodon* and other species of Helaletidae [5]. However, the cheek teeth of *Teleolophus* and *Deperetella* have been suggested to have a compound HSB configuration with a transverse HSB in an inner zone [5, 21], which likely derived from the vertical HSB in *Irenolophus*. The proposal of the origin of vertical HSB in *Irenolophus* enamel from the curved HSB is tentative, and needs to be tested by phylogenetic analysis.

## Discussion

### Origin of deperetellids

When Matthew and Granger [11, 25] first described *Teleolophus* and *Deperetella*, they considered the two genera to be clearly related to *Colodon*, and assigned them to Helaletidae. This taxonomy was followed by Simpson [26] and Gromova [27]. However, regarding the similarities between deperetellids and helaletids as convergence, Radinsky [1] erected a new family Deperetellidae, which consists of five genera: *Teleolophus* and *Pachylophus* with submolariform or non-molarifrom premolars [1, 28]; and *Deperetella*, *Diplolophodon*, and *Bahinolophus* with molarized premolars [1, 2, 29]. Later, other workers considered either Rhodopagidae or Lophialetidae as the sister group to Deperetellidae [10, 30]. Dashzeveg and Hooker [31] erected *Irdinolophus*, based on “*Helaletes*” *mongoliensis*, as a representative of primitive deperetellids. However, Bai et al. [16] reassigned “*Helaletes*” *mongoliensis* to the helaletid *Desmatotherium* as originally named by Osborn [32]. Based on the postcranial material of *Teleolophus* from the Erlian Basin, Bai et al. [6] argued that *Colodon* is more similar to *Heptodon* than to *Teleolophus*.

The origin of deperetellids has long been obscured because of their peculiar inverted U-crest formed by the protoloph, paracone, and metaloph, and highly reduced metacone on the upper molars. Compared with the basal ceratomorph *Homogalax*, *Irenolophus* mainly resembles *Helaletes* and other more derived helaletids [12] in having the reduced, flat, lingually depressed metacones on the upper molars, and the trend towards the bilophodonty on the lower molars. The wider talonids on p2-p4 are reminiscent of those of the helaletid *Desmatotherium intermedius* and *Colodon* [12]. However, the lower premolars of *Colodon* and *Desmatotherium intermedius* are relatively shorter with distinct entocoinds on p3-p4. Furthermore, helaletids except for *Heptodon* differ from *Irenolophus* in the lack of p1, and in having a deep, wide narial notch without naso-premaxilla contact. However, the skull of *Heptodon posticus* [7] is similar to that of *Irenolophus* in having the following characters: a relatively shallow narial notch with the premaxilla in contact with the nasal; the posterior border of the palate situated between the posterior border of M2s; a sharp sagittal crest; an oval foramen separated from the middle lacerate foramen; and the symphyseal region of the mandible extending posteriorly to p1 with a deeply concave dorsal surface that rises anteriorly. The skull of *Heptodon* mostly differs from that of *Irenolophus* in having a more posteriorly retracted narial notch to the point over the postcanine diastema (closer to P1 than the canine), a more pronounced postorbital constriction, and an infraorbital foramen situated above P3 in a relatively higher position. Thus, these mosaic craniodental characters suggest that *Irenolophus*, as well as the deperetellids, probably also derive from some *Heptodon*-like helaletids, and they evolved towards a bilophodont configuration. They would have diverged from *Helaletes* and other more derived helaletids as early as late early Eocene.

The hypothesis that *Irenolophus* probably derived from basal Helaletidae is also supported by its shared carpal characters with that of *Heptodon* [7]: the scaphoid with the proximal and distal lunar facets converging posteriorly; the posterior magnum facet posteriorly extended (but more distally directed than in *Heptodon*); the lunar relatively long anteroposteriorly with a roughly rectangular outline in lateral view, a slightly concave medial border of the radial facet, the anterior magnum facet relatively large and vertical, and the unciform facet directed nearly horizontally; the magnum with a nearly horizontal anterior scaphoid facet, a relatively large, nearly vertical anterior lunar facet, and a large, vertically placed Mc II fact; and the unciform with a short posterior process and a relatively large Mc V facet, indicating the Mc V is not highly reduced. On the other hand, the carpals of *Irenolophus* resemble those of *Teleolophus* and *Deperetella* in some other features (discussed above), which in turn are different from those of *Heptodon*. However, the proposal that deperetellids originated from basal helaletids needs to be tested by phylogenetic analysis. It is necessary to mention that the conventional ‘Helaletidae’ is not a monophyletic group because it does not include the monophyletic Tapiridae, which has been considered to have evolved from *Helaletes* with *Plesiocolopirus* as a transitional form [12]. However, Colbert [8] considered *Tapirus* as more closely related to *Colodon* than to *Protapirus*, based mainly on cranial features. The ‘Helaletidae’, Tapiridae, and Deperetellidae are closely related. The latter two families probably originated from the ‘Helaletidae’, but their specific phylogenetic relationships remain unclear and will be investigated by future analyses. Our preliminary phylogenetic analysis of Ceratomorpha based on a relatively large data matrix support that both tapirids and deperetellids derived from helaletids as suggested by the morphologic comparisons.

Recently described, the early Eocene *Meridiolophus* from the Sanshui Basin of Guangdong Province, China [33] and *Vastanolophus* from the Vastan Mine of India [34] represent basal taxa of ‘Helaletidae’ and/or intermediate groups between *Homogalax*-like taxa and *Heptodon*. Their morphologies support the hypothesis that tapiroid perissodactyls, including basal ‘helaletids’, originated in Asia.

### Patterns of premolar molarization in perissodactyls

**Fig 7:**
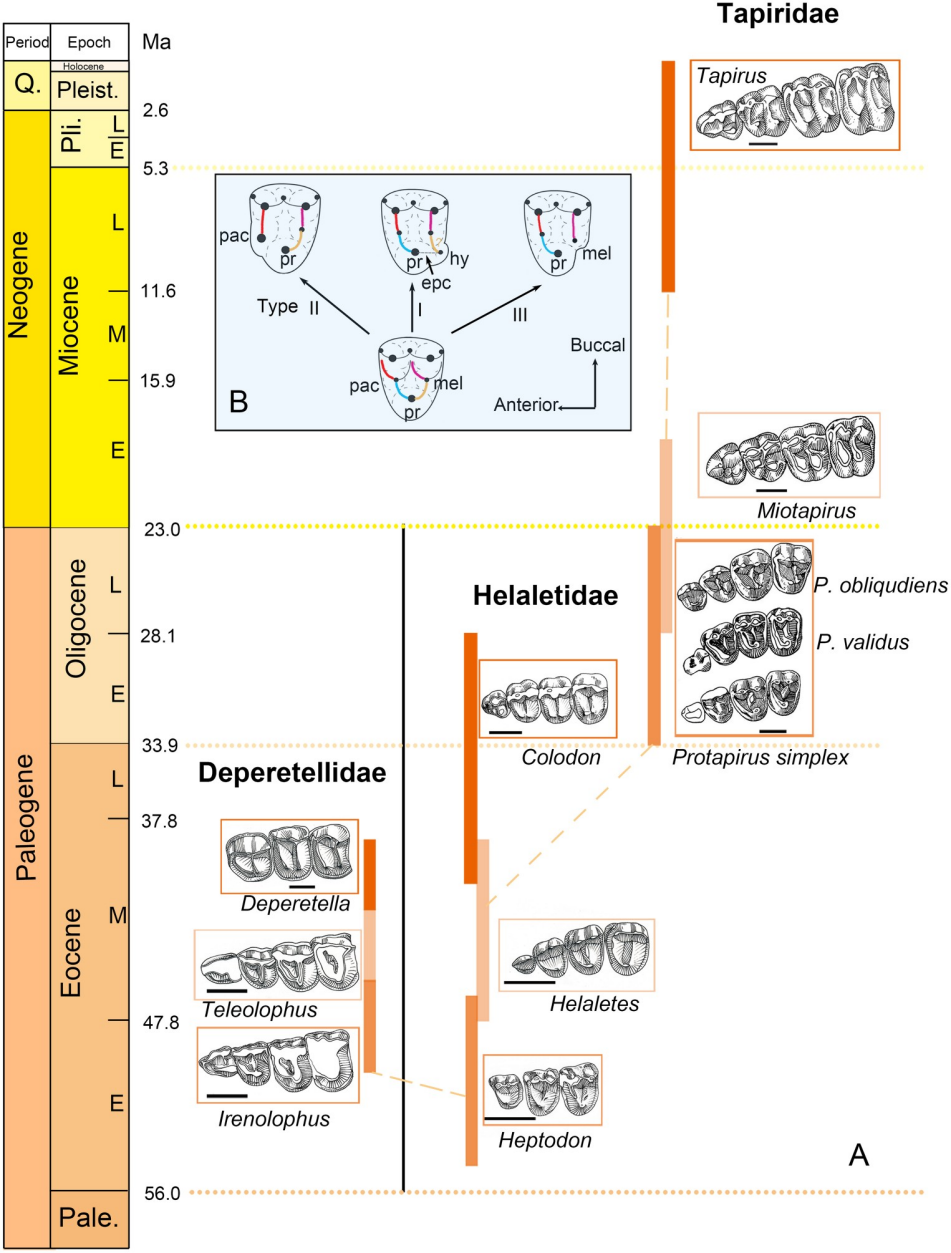
The gradational changes of premolars in tapiroid lineages with different patterns of the premolar molarization. (A) The hypothesized phylogenetic relationships of Tapiroidea [12] with deperetellids added, showing the premolars of tapiroids gradually molarized through different patterns over time. The premolars in Tapiridae modified from Schlaikjer [40] (Museum of Comparative Zoology, Harvard University). (B) The three patterns of premolar molarization in perissodactyls modified from Holbrook [38]. All premolar series are shown from the left side, and the last tooth is P4. Abbreviations: pac, paraconule; pr, protocone; epc, endoprotocrista; hy, hypocone; and mel, metaconule. Scale bar = 10 mm.

Similar to the emergence of the molar hypocone in ungulates [35], molarization of premolars also probably plays an important role in perissodactyl diversity and adaption [36]. Perissodactyls have a variable degree of molarization of premolars, and there is a trend from non-molariform towards completely molariform teeth [15]. However, different individuals in the same species may display variable degrees of molarization because of intraspecific variation [37]. Holbrook [38] figured out two modes of premolar molarization in perissodactyls. In perissodactyls, an endoprotocrista (which usually extends posteriorly rather than posterobuccally as in postprotocrista from the protocone) was considered as playing an important role in the molarization [38]. The hypocone is usually developed from the endoprotocrista, and separated from the protocone by a lingual groove. This pattern (here assigned as Type I) is the most common in perissodactyls as observed in helaletids, tapirids, *Chasmotherium*, most rhinocerotoids, and palaeotheres [15, 38] (Fig 7A). Another pattern of premolar molarization involves the enlargement and lingual migration of the paraconule, which was considered only known from P3 of early equids [38, 39].

The moderately worn P3 of *Irenolophus* from the late early Eocene shows some unique features among tapiroids, and indicates a different pattern of premolar molarization in perissodactyls (Fig 7A). On the lingual side of P3, there is only one main cusp, which is interpreted as the protocone rather than the hypocone based on its position and relatively large size. Moreover, if the lingual cusp were interpreted as the hypocone, the corresponding talonid of p3 would have a well-developed entoconid [36], which is absent in the species. The metaloph is strong and straight, and the protoloph is posterolingually extended with a swollen paraconule in the middle, joining the protocone by a narrow ridge a short distance to the lingual end of the protocone. On the anterolingual side of the protocone, there is a shallow groove, which demarcates the separation between the protocone and the paraconule. The morphology of P4 in *Irenolophus* is similar to that of P3, although the former is heavily worn. From *Irenolophus* to the early middle Eocene (Irdinmanhan) *Teleolophus*, the groove between the protocone and the paraconule became deeper and more distinct, although the “protoloph” still contacts the metaloph. However, the protoloph and the metaloph are sometimes variable in forming a complete loop on upper premolars of *Teleolophus* [28]. The protocone and the paraconule show a greater degree of merging with the lophs in *Teleolophus* than in *Irenolophus*. In the late middle Eocene (Sharamurunian) *Deperetella*, P3–4 are completely molariform with a parallel “protoloph” and metaloph. The anterolingual cusp of P2–4 is homologous with the paraconule, and the posterolingual cusp is homologous with the protocone rather than with the hypocone. A similar pattern of premolar molarization by means of the separation between the paraconule and the protocone (assigned to Type II) is also present in P3 of Eocene horses from “*Hyracotherium*” to *Orohippus* [38, 39] and P2 of the hyrachyid *Metahyrachyus* [41]. Butler [36], however, considered the homologies of their lingual cusps as the hypocones instead of the protocones. By contrast, the P4 molarization was formed by the emergence of the hypocone in the Eocene equids [39], suggesting different premolar loci could possess different patterns of premolar molarization in the same lineage. It is noteworthy that the European middle Eocene to early late Eocene *Chasmotherium cartieri* also possesses precocious molariform premolars [42] as in *Deperetella*, but that occurrence is attributed to parallel evolution.

Amynodontid rhinocerotoids (also known as “aquatic rhinoceros”) display the third pattern of premolar molarization. In the primitive early middle Eocene (early Uintan) *Amynodon*, P3 and P4 have a single lingual cusp (protocone), and the metaconule (or metaloph) joins the protocone at the base or in a high position [43]. In the late middle Eocene (Duchesnean) *Megalamynodon* [44], the P3–4 protoloph and the metaloph are widely separated lingually by the breakage between the protocone and the metaconule, although the protoloph is much stronger and more lingually extended than the metaloph. In the late middle Eocene *Huananodon* [45], the P2–3 protoloph and metaloph are parallel and extended equally lingually. This pattern of premolar molarization by the separation between the protocone and the metaconule (assigned to Type III) is currently only known in amynodontids among perissodactyls (Fig 7B). Similarly, metaconule-derived pseudohypocones on the upper molars have been proposed in Artiodactyla, Pleuraspidotheridae, and Paenungulata [35, 46, 47].

The main difference among the different types of premolar molarization patterns in perissodactyls is that the type I was formed by the emergence of hypocone, but the other two types were initiated by the deformation of already existing ridges and cusps (Fig 7B). The different patterns of premolar molarization in perissodactyls indicate that the various mechanisms controlled by genes, which play an important role in the development of cusps and crests as demonstrated in experiments on rodents *in vivo* [48], have affected the evolutionary history of perissodactyls since the Eocene. The hypothesis that homeobox tooth gene could account for the molarization of the premolars has not been tested [49], and further investigation on this issue is needed.

## Conclusions

The primitive tapiroid *Irenolophus qii* gen. et sp. nov. from the late early Eocene of China provides insight on the origin of the endemic Asian deperetellids. Based on craniodental and carpal characters, we propose that deperetellids probably arose from *Heptodon*-like helaletids, but the specific relationships among ‘Helaletidae’, Tapiridae, and Deperetellidae need detailed phylogenetic analysis. The HSB of *Irenolophus* enamel is characterized by a vertical configuration, which probably originated from curved HSB such as present in *Heptodon* and other species of Helaletidae [5]. Furthermore, we synthesize three different patterns for the molarization of the premolars in perissodactyls based on the morphology of *Irenolophus* and comparisons with other ceratomorphs. The pattern of molarization in deperetellids is characterized by the paraconule and protocone separating, which is different from the premolar hypocone emerging from the endoprotocrista in most ceratomorphs and palaeotheres, or the metaconule-derived pseudohypocone in amynodontids.

## Supporting information

S1 Table Measurements of teeth of *Irenolophus* and other deperetellids (Fig 2) (in mm). (XLSX)
